# Biodegradation of reactive textile dye Novacron Super Black G by free cells of newly isolated *Alcaligenes faecalis* AZ26 and *Bacillus* spp obtained from textile effluents

**DOI:** 10.1016/j.heliyon.2019.e02068

**Published:** 2019-07-11

**Authors:** Md. Zobaidul Hossen, Md. Eleus Hussain, Al Hakim, Kamrul Islam, Md. Nizam Uddin, Abul Kalam Azad

**Affiliations:** a Department of Genetic Engineering and Biotechnology, School of Life Sciences, Shahjalal University of Science and Technology, Sylhet, 3114, Bangladesh; b Department of Chemistry, School of Physical Sciences, Shahjalal University of Science and Technology, Sylhet, 3114, Bangladesh

**Keywords:** Biotechnology, Microbiology, Biodegradation, Reactive dye, Decolorization, Bacterial isolate, Novacron Super Black G

## Abstract

Bacteria were isolated from effluents of textile industries and screened by their capability to decolorize at least one of eight reactive dyes used in the textile industries. Three isolates having the capability to decolorize the highest number of dyes with more than 25% of decolorization were identified as *Alcaligenes faecalis* AZ26, *Bacillus cereus* AZ27 and *Bacillus* sp. AZ28 based on morphological, cultural, biochemical characteristics, and 16S rDNA sequence analysis. The decolorization capability of these three bacterial isolates was optimized under different physicochemical conditions by using Novacron Super Black G (NSB-G), one of the eight reactive dyes commonly used in textile industries. These bacterial isolates grew well in the presence of up to 500 mg L^−1^ of NSB-G and showed decolorization of approximately 90% at 200 mg L^−1^ of NSB-G after 96 h of cultivation at 37 °C and pH 8.0 under static condition. Decolorization of NSB-G by the bacterial isolates was investigated using UV-VIS spectrophotometry and Fourier Transform Infrared Spectroscopy (FTIR) analysis. The UV-visible absorbance spectra and the FTIR spectrum of the decolorized NSB-G significantly differed from those of the parent dye, indicating that the NSB-G was degraded by the bacterial isolates. High decolorization extent supports the notion that the bacterial isolates reported herein might have potential in the biological treatment of dyeing mill effluents.

## 1 Introduction

Textile, pharmaceutical, cosmetic, paper and food industries use synthetic dyes widely (Pandey et al., 2007; Kant, 2012). Usually synthetic dyes are more stable against biodegradation due to having complex aromatic molecular structures (Aksu, 2005; Dellamatrice et al., 2017). About 10,000 different dyes and pigments are used in textile industries and over 7 × 10^5^ tons are produced worldwide per annum (Aksu and Tezer, 2005; Daneshvar et al., 2007; Celia and Suruthi, 2016). The usage of dyestuff is increasing tremendously due to the rapid increase of industrialization and man's urge for color (Mohan et al., 2002). Due to their wide variety of color shades, high wet fastness profiles, ease of application, brilliant colors and minimal energy consumption, reactive dyes are widely used in the textile industries (Shah et al., 2013). There are three common groups of reactive dyes: Azo, anthraquinone and phthalocyanine (Axelsson et al., 2006), most of which are toxic, carcinogenic and mutagenic (Acuner and Dilek, 2004; Rauf and Salman Ashraf, 2012; Stiborova et al., 2013). Improper discharge of highly colored effluent containing reactive dyes causes damage to the aquatic environment. Due to the presence of aromatics, metals, chlorides, etc., reactive dyes may be toxic to some aquatic life and may significantly affect photosynthetic activity in aquatic phototrophs because of reduced light penetration (Celia and Suruthi, 2016). Reactive dyes have high tinctorial value and less than 1 ppm of the dye produces obvious coloration (Gupta et al., 2003).

For the removal of dyes from wastewater, various physical and chemical methods such as adsorption, coagulation–flocculation, oxidation and electrochemical methods can be used (Lin and Peng, 1994). But these methods have many drawbacks in terms of high-energy costs, high-sludge production, and formation of by-products (Sarioglu et al., 2007; Celia and Suruthi, 2016). Conversely, being cost saving and environmentally benign, bioprocessing can overcome these demerits (Kurade et al., 2017).

In the recent years, a number of studies have focused on using some microorganisms (Chen et al., 2003; Acuner and Dilek, 2004; Mishra and Malik, 2014; Shen et al., 2015; Kurade et al., 2017; Veena et al., 2019) for degrading and absorbing dyes from wastewater. A wide variety of microorganisms including bacteria, fungi, yeast and algae are able to decolorize and degrade a wide range of dyes (Ayed et al., 2010; Kabra et al., 2011; Patel et al., 2013; Saratale et al., 2013; Veena et al., 2019). Under certain conditions, bacteria can rapidly degrade and even completely mineralize many reactive dyes (Chen et al., 2003; Asad et al., 2007; Jadhav et al., 2011; Kurade et al., 2012; Barapatre et al., 2017). The intermediate metabolites such as aromatic amines, generated during the decolorization process, can be degraded by the hydroxylase and oxygenase produced by bacteria (Pandey et al., 2007; Wanyonyi et al., 2017).

Bangladesh has emerged as one of the largest garment-manufacturing nations in the world. The garment sector has become the largest sector of the country's foreign exchange earnings and employs about 50% of its industrial work force (Farhana et al., 2015; Shuchismita and Ashraful, 2015). The textile industries use large quantities of reactive dyes in their production processes and discharge waste water into sewers and drains without any treatment (Chindah et al., 2004). The physicochemical parameters of the effluents in Bangladesh are much higher than the standard value recommended by Department of Environment (Shuchismita and Ashraful, 2015). The presence of reactive dyes in surface and subsurface water is making them not only aesthetically objectionable but also causing many human health hazards resulting in diseases, viz. mucous membrane, dermatitis, perforation of nasal septum and severe irritation of respiratory tract and toxicological effects as well as allergenic potential (Islam et al., 2011; Yadav, 2014; Rovira and Domingo, 2019). Textile effluents are spreading in the water body and impart a chemical load to the environment; its integrity renders the environmental quality fairly deplorable affecting aquatic biodiversity and plant growth. Because of this, people living near textile industries are now being threatened due to the environmental degradation (Sultana et al., 2009). Therefore, a sustainable bioprocess is badly needed to remedy the toxicity imparted by the reactive dyes in the untreated textile effluents. In this regard, achievement of the textile reactive dyes-degrading bacteria from the indigenous environment is very important. Bacteria present in the untreated textile effluents might have capabilities to degrade textile reactive dyes. Although several studies have been done showing absorption and degradation of dyes by microorganisms (Mishra and Malik, 2014; Shen et al., 2015; Kurade et al., 2017; Veena et al., 2019), additional studies are required to develop biotechnology to degrade and detoxify the reactive dyes in effluents and wastewaters generated from textile industries.

In the present study reported herein, bacteria were isolated and identified from untreated textile effluents. These isolates decolorized eight reactive dyes used in the textile industries. Different physicochemical parameters were optimized for decolorization of Novacron Super Black G (NSB-G), one of the eight reactive dyes commonly used in textile industries, and bacterial biodegradation was shown as the mechanism of decolorization of NSB-G.

## 2 Materials and methods

### 2.1 Chemicals

To obtain bacterial isolates with a high decolorizing capability, eight textile reactive dyes—namely Novacron Yellow S3R (NY-S3R), Novacron Blue SGL (NB-SGL), Novacron Ruby S3B (NR-S3B), Novacron Navy FNBN (NN-FNBN), Novacron Super Black G (NSB-G), Novacron Turquise HGN (NT-HGN), Novacron Dark Blue WR (NDB-WR), Novacron BR Blue FNG (NBRB-FNG)—were collected from a textile industry located at Kalarpool, Patia, Chittagong, Bangladesh. NSB-G, one of the eight reactive dyes commonly used in textile industries, was selected to optimize physicochemical parameters for decolorization and to elucidate the mechanisms of decolorization. All chemicals were purchased from Sigma Aldrich, India unless noted.

### 2.2 Isolation, screening and identification of dye decolorizing bacteria

Untreated effluent samples were collected in sterile vials from the sites of four textile industries located at Kalarpool, Patia and Oxygen Circle in Chittagong, Bangladesh. Samples were transported immediately to the laboratory and used in the experiment. Effluent samples diluted up to 10^4^ times were cultured separately on the nutrient agar plate as described previously (Azad et al., 2009). From nutrient agar plates, 30 bacterial colonies were randomly selected for obtaining pure culture. Primary screening for decolorization capability of these bacterial isolates was carried out for 48 h using the eight reactive dyes. One loopfull of each bacterial isolate was inoculated in 10 ml sterile nutrient broth (glucose, 1 gm L^−1^; peptone, 1 gm L^−1^; beef extract, 1 gm L^−1^ and yeast extract, 1 gm L^−1^, pH 7.0) containing any of the eight dyes in the test tubes at a concentration of 200 mg L^−1^. Nutrient broth containing peptone (partially digested protein), beef and yeast extracts is a complex medium that meets energy, carbon, nitrogen, Sulphur, soluble vitamins and minerals requirements to support the growth of most heterotrophic bacteria (Tortora et al., 2010). For screening dye decolorizing bacteria, usually 100–300 mg L^−1^ dye is used (Lalnunhlimi and Krishnaswamy, 2016). Isolates that decolorized at least one of these eight dyes within 48 h were taken for further screening (up to seven days). During screening, dye decolorizing isolates were preliminary identified on the basis of morphological, cultural and biochemical characteristics according to Bergey's Manual of Systematic Bacteriology (Staley et al., 2001).

### 2.3 16S rDNA gene sequencing

The genomic DNA extraction of bacterial isolates was done by using Favorgen Cultured Cell Genomic DNA Extraction Kit in accordance with the manufacture's instruction (Favorgen Biotech Corporation, Taiwan). Polymerase chain reaction (PCR) was carried out in a thermo cycler SimpliAmp TM (Applied Biosystems; USA). The 16S rDNA of the bacterial isolates was amplified by using a universal 16S rDNA specific primer pair 5′- CGG TTA CCT TGT TAC GAC TT-3′ (forward) and 5′- CGG TTA CCT TGT TAC GAC TT- 3′ (reverse) (Iqbal et al., 2018). Amplification reactions were executed with 2x G2 hot start colorless master mix (Promega, Madison, WI, USA) as described previously (Hakim et al., 2018). The thermocycler was programmed for 1 cycle at 94 °C for 2 min; 35 cycles at 94 °C for 30 s, at 55 °C for 30 s and at 72 °C for 2 min; 1 cycle at 72 °C for 10 min. PCR products were purified from agarose gel by an extraction kit (ATP^TM^ Gel/PCR Extraction kit, ATP Biotech Inc., Taiwan) and sequenced by a DNA sequencer (Model 3130, ABI Automated Genetic Analyzer, Hitachi, Japan).

The 16S rDNA sequences were analyzed using Chromas 2.6.2 (https://chromas.software.informer.com/2.6/). The sequences were searched for similarities in the BLAST (https://blast.ncbi.nlm.gov/Blast.cgi) search program and aligned with the similar sequences by using Clustal Omega (https://www.ebi.ac.uk/Tools/msa/clustalo/). The phylogenetic tree was constructed using Molecular Evolution Genetic Analysis (MEGA), Version 5.0 (Tamura et al., 2011) as described previously (Azad et al., 2016, 2018).

### 2.4 Measurement of decolorization extent

A 5% (v/v) inoculum of a bacterial isolate freshly cultured for 18 h was inoculated into sterile nutrient broth containing each reactive dye of specified concentration in 50 ml conical flask separately. The reaction was carried out at 37 °C under static condition for a period as noted. Culture (2 ml) from the nutrient broth containing the dye was collected and centrifuged at 8000 rpm for 10 min to remove bacterial cells. The percent of decolorization of the supernatant was determined by measuring the decrease of the absorbance at the corresponding λ_max_ of each dye at a definite time interval during the decolorization process using a UV-visible spectrophotometer (Shimadzu UV-1800, Japan). The λ_max_ of NY-S3R, NB-SGL, NR-S3B, NN-FNBN, NSB-G, NT-HGN, NDB-WR and NBRB-FNG was 420, 620, 545, 597, 600, 623, 635 and 598 nm, respectively. Decolorization extent was calculated using the following equation:

$$\text{Decolorization}\;\text{extent}\;\text{(\%)}=\frac{\text{OD}1-\text{ODt}}{\text{OD}1}\times 100$$

Where, OD_1_ refers to the initial absorbance before decolorization, OD_t_ refers to the absorbance after decolorization.

### 2.5 Effects of different physicochemical parameters on decolorization of NSB-G

The effects of different physicochemical parameters on the decolorization of reactive dye by bacterial isolates were investigated by using NSB-G. To investigate the effects of initial dye concentration, media containing 50, 100, 200, 500 and 1000 mg L^–l^ dye were subjected to decolorization for 96 h. To study the time course, media containing 200 mg L^−1^ dye was subjected to decolorization up to 96 h. In the both cases, pH of the media was adjusted to 7.0 and the experiment was conducted at 37 °C. The extent of dye decolorization was observed every 24 h interval. To obtain the optimum temperature for dye decolorization, investigation was carried out at 22, 30, 37 and 45 °C, and the initial pH of the media was 7.0. To observe the effects of different initial pH on the decolorization, media were adjusted to different pH as indicated in Fig. 4. The culture was carried out at 37 °C. Experiments were performed in 15 ml glass tubes containing 10 ml nutrient broth medium and 5% (v/v) inoculum (∼6×10^6^ bacterial cells) without agitation. The number of bacterial cells in 5% inocula was predicted by OD_600_, which was routinely verified by total viable bacterial count on nutrient agar.

### 2.6 UV-visible and Fourier Transform Infrared Spectroscopy analysis for biodegradation of NSB-G by bacterial isolates

In order to investigate the decolorization manner of NSB-G by bacterial isolates, nutrient broth containing NSB-G was inoculated either with autoclaved bacterial culture (heat treated) or untreated bacterial culture. After 24, 48 and 96 h of incubation at 37 °C, bacterial cultures were centrifuged at 8000 rpm for 10 min and the supernatant was scanned from 200 nm to 800 nm using a UV-visible spectrophotometer (Shimadzu UV-1800, Japan) to investigate the presence of new compounds in the medium. The initial pH of nutrient broth media was adjusted to 7.0.

The infrared spectra of dyes and their metabolites were recorded on KBr pellets with a SHIMADZU IR spectrometer (Model: Prestige 21). Degraded products were extracted by ethyl acetate. Solvent was removed by rotary evaporator and dried under vacuum. Then Fourier Transform Infrared Spectroscopy (FTIR) spectra of pure dyes and their degraded products were recorded on KBr pellets (Ayed et al., 2011).

### 2.7 Statistical analysis

Student's *t* test was used for statistical analysis. A *P* value of <0.05 was considered as statistically significant. Data were presented as the means ± standard errors of the means (SEM) of repeated experiments (n = 5), or as noted in the figure legends.

## 3 Results and discussion

### 3.1 Isolation and screening of reactive dye decolorizing bacterial isolates

Cultivable bacteria from the effluent of textile industries were isolated by using a complex nutrient agar media. Thirty heterotrophic bacterial isolates were randomly selected from the nutrient agar plate for investigation of their capability to decolorize the reactive dyes. Primary screening for dye decolorization of the 30 bacterial isolates was carried out in nutrient broth for 48 h by using eight reactive dyes (200 mg L^−1^) separately (Table 1). Fourteen isolates that decolorized at least one of these eight dyes were taken for further screening (up to seven days). Decolorization extent and number of decolorizing isolates increased with further incubation after 48 h (data not shown). Morphological, cultural and biochemical characteristics indicated that these fourteen bacterial isolates were distributed among the bacterial genera of *Aeromonas* (1 isolate)*, Alcaligenes* (1 isolate)*, Bacillus* (4 isolates)*, Citrobacter* (1 isolate)*, Morganella* (1 isolate)*, Pseudomonas* (4 isolates) and *Serattia* (2 isolates)*.* The morphological and biochemical characteristics that were used for their preliminary identification are summarized in Table 2. For further study, we selected isolates 26 (*Alcaligenes*), 27 (*Bacillus* sp) and 28 (*Bacillus* sp) that predominantly decolorized reactive dyes during screening. Isolate 2 showed decolorization of five dyes; however, it was not selected for further study since its decolorization extent of NSB-G was lower than 25%. Although many bacterial species have been shown to degrade dyes, *Bacillus, Alcaligens* and *Pseudomonas* species are reported as potential reactive dye degraders (Jadhav et al., 2008; Kalyani et al., 2008; Shah et al., 2012, 2013; Wang et al., 2013; Celia and Suruthi, 2016; Wanyonyi et al., 2017).

**Table 1:** Screening of dye degrading bacteria following incubation at 37 °C for 48 h.

Dye	Bacterial isolates																													
	1	2	3	4	5	6	7	8	9	10	11	12	13	14	15	16	17	18	19	20	21	22	23	24	25	26	27	28	29	30
NY-S3R	-	+	-	-	-	-	-	-	-	-	-	-	-	-	-	-	-	-	-	-	-	-	-	-	-	-	+	+	-	-
NB-SGL	+	+	-	-	-	-	-	-	-	-	-	-	-	+	-	+	-	+	-	-	-	-	-	-	-	+	+	+	-	-
NR-S3B	-	+	-	-	-	-	-	-	-	-	-	-	-	-	-	-	-	-	-	-	-	-	+	-	+	-	+	+	-	-
NN-FBN	-	+	-	-	-	-	-	-	-	-	-	-	-	-	-	-	-	-	-	-	-	-	+	-	-	+	+	+	-	-
NSB-G	-	-	-	-	-	-	-	-	-	-	-	-	-	-	-	-	-	-	-	-	-	-	-	-	-	+	+	+	+	-
NT-HGN	-	-	-	-	-	-	-	-	-	-	-	-	-	-	-	-	-	-	-	-	-	-	-	-	-	-	-	-	-	-
NDK-WR	-	+	+	-	-	-	-	-	-	-	-	-	+	+	-	-	-	-	-	-	-	-	+	-	-	+	+	+	+	-
NBRB-FNG	-	-	-	-	-	-	-	-	-	-	-	-	-	-	-	-	-	-	-	-	-	-	+	+	-	+	+	+	-	-

+, Presence of decolorization.

-, Absence of decolorization.

**Table 2:** Morphological and biochemical characteristics of bacterial isolates 26, 27 and 28.

Morphological and Biochemical tests	Bacterial isolates		
	Isolate 26	Isolate 27	Isolate 28
Shape	Rod	Rod	Rod
Motility	+	+	+
Catalase production	+	+	+
Oxidase production	+	+	+
Gram staining	-	+	+
Indole test	-	-	-
Methyl red test	-	+	+
Voges-Proskauer test	-	-	-
Citrate utilization	+	-	-
Triple Sugar Iron Agar test (Slant/Butt)	R/R^a^	K/A^b^	K/A
Maltose fermentation	-	+	+
Lactose fermentation	-	-	-
Glucose fermentation	-	+	+
Spore test	+	+	+
Bacterial genus	*Alcaligenes*	*Bacillus*	*Bacillus*

a R/R, Orange red.

b K/A, Red/Yellow.

### 3.2 Identification of bacterial isolates based on 16S rDNA sequence

**Fig. 1:**
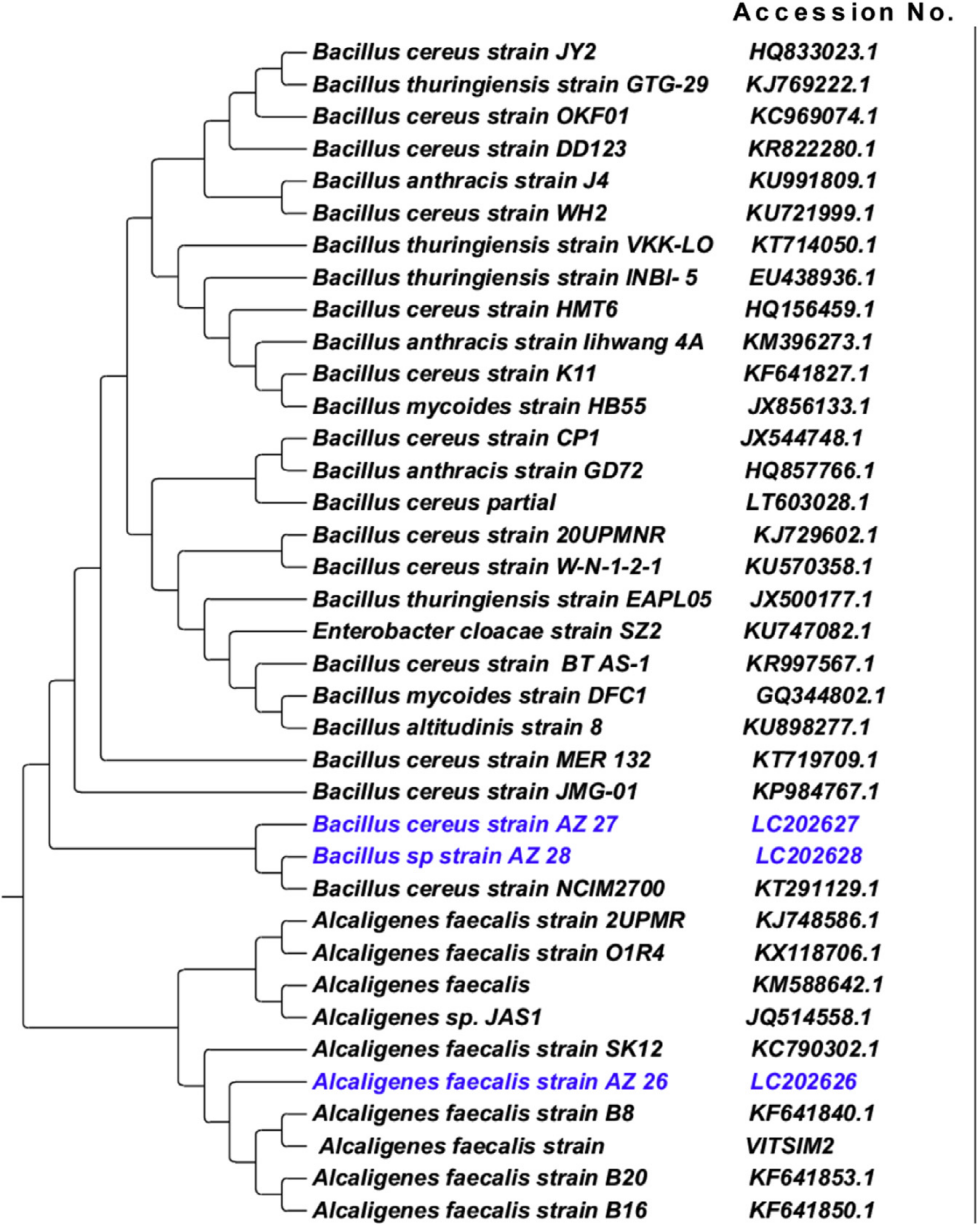
Phylogenetic relationship of *Alcaligenes faecalis* AZ26 (isolate 26), *B. cereus* AZ27 (isolate 27) and *Bacillus* sp. AZ28 (isolate 28) with other *Alcaligenes* and *Bacillus* species.

The 16S rDNA gene sequencing was performed for molecular identification of the three bacterial isolates. PCR amplicons of genomic DNA of the three bacterial isolates were approximately 1550 base pair. The 16S rDNA sequences of bacterial isolates 26, 27 and 28 were deposited to DNA Data Bank, Japan (DDBJ) with the DDBJ, EMBL and Gene Bank accession no. LC202626, LC202627 and LC202628, respectively. BLAST similarity search showed that the bacterial isolate 26 was similar to *Alcaligenes* and isolates 27 and 28 were similar to *Bacillus*. A phylogenetic tree constructed with the similar sequences showed that isolates 26, 27 and 28 were closely related to *Alcaligenes faecalis*, *Bacillus cereus* and *Bacillus* sp., respectively (Fig. 1) and were named as *Alcaligenes faecalis* AZ26, *Bacillus cereus* AZ27 and *Bacillus* sp. AZ28.

### 3.3 Decolorization of various textile reactive dyes

**Fig. 2:**
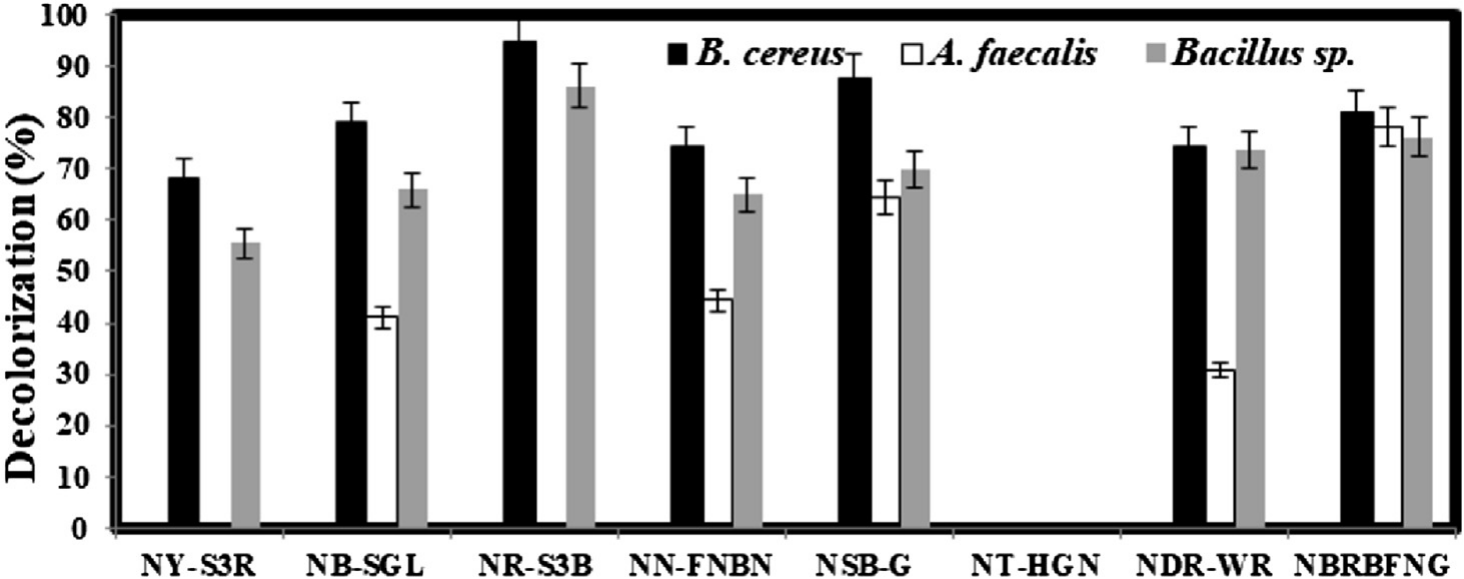
Decolorization of various textile dyes after 48 h of incubation at 37 °C. The initial pH of the nutrient broth was adjusted to 7.0 and the inoculum size and dye concentration was 5% and 200 mg L^−1^, respectively.

Out of the eight reactive dyes at a concentration of 200 mg L^−1^, seven dyes were efficiently decolorized by *B. cereus* AZ27 and *Bacillus* sp. AZ28 after 48 h of cultivation (Fig. 2). A maximum decolorization extent of approximately 95 and 85% was recorded for *B. cereus* AZ27 and *Bacillus* sp. AZ28*,* respectively when Novacron Ruby S3B was used. *B. cereus* AZ27 and *Bacillus* sp. AZ28 decolorized six other dyes ∼65–85% and ∼55–75%, respectively except Novacron Turquise HGN that was not decolorized at all by any of the three bacterial isolates. On the other hand, *A. faecalis* AZ26 decolorized five dyes and maximum decolorization (∼78%) was obtained with NBRB-FNG. The variation in the decolorization of different reactive dyes might be attributable to the structural diversity of the dyes and type of bacterial strains (Kalyani et al., 2008).

### 3.4 Effects of incubation period and initial dye concentration on the decolorization of NSB-G

**Fig. 3:**
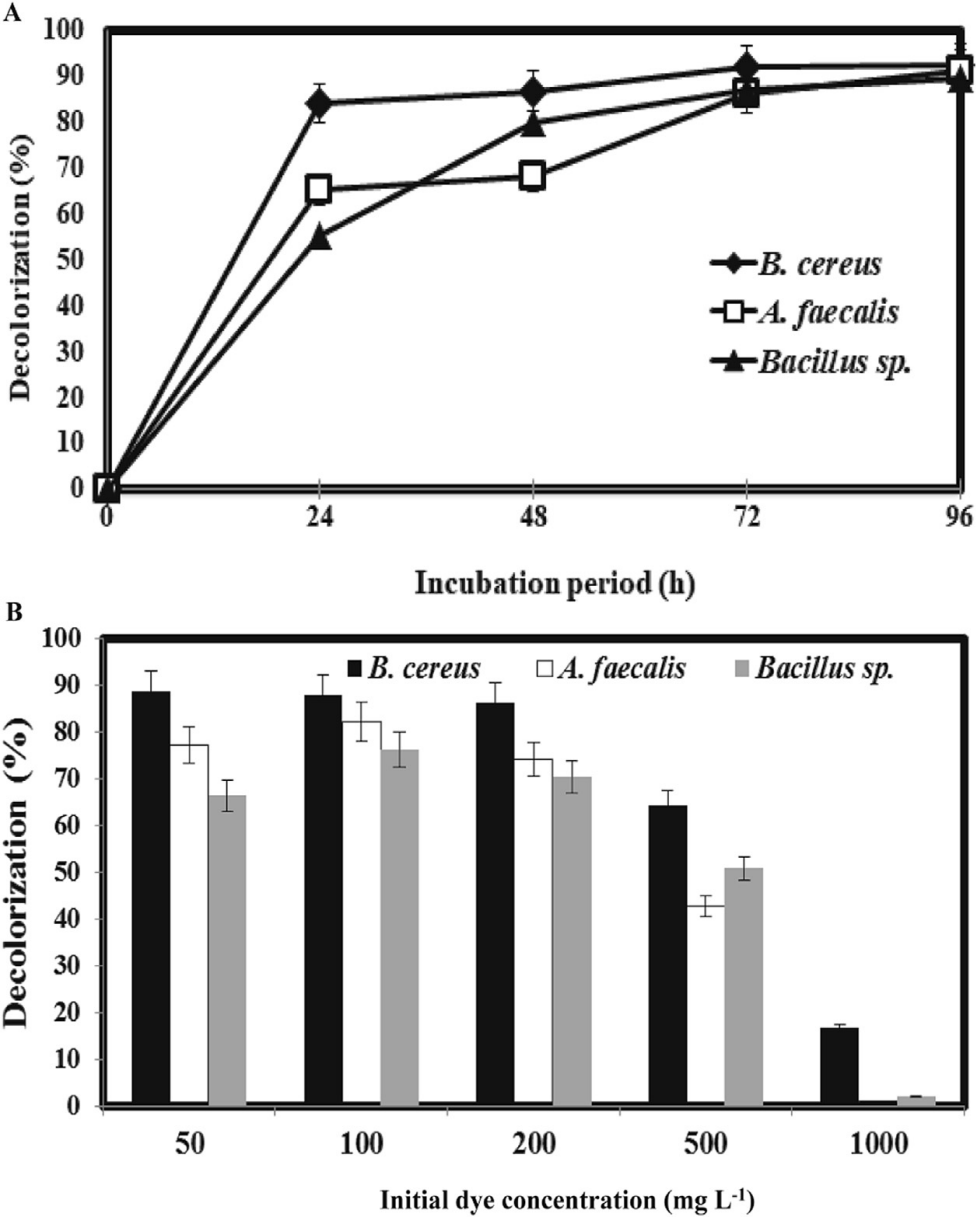
Effects of incubation period (A) and initial dye concentration (B) on decolorization of NSB-G. The concentration of NSB-G in (A) was 200 mg L^−1^. The decolorization extent was measured after 96 h of cultivation at 37 °C with the initial pH 7.0 and 5% inoculum.

Time course study revealed that the decolorization of NSB-G by the three bacterial isolates was almost saturated (∼90%) after 72 h of incubation (Fig. 3A). However, decolorization of NSB-G by *B. cereus* AZ27 increased sharply to achieve more than 80% of the total decolorization after 24 h. On the other hand, decolorization of NSB-G by *A. faecalis* AZ26 and *Bacillus* sp. AZ28 after 24 h was only about 65% and 55%, respectively.

**Fig. 4:**
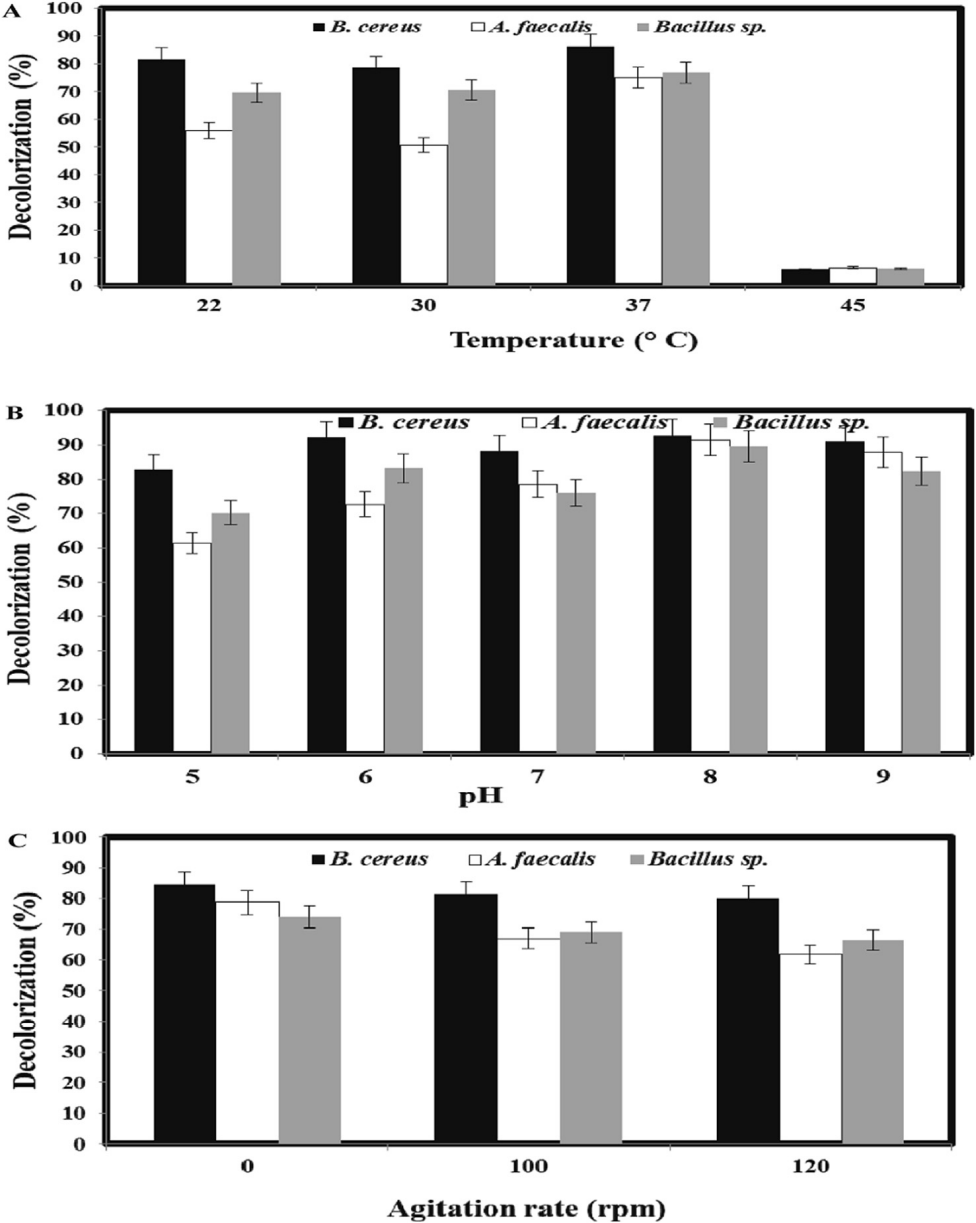
Effects of temperature (A), pH (B) and agitation (C) on decolorization of NSB-G. The initial pH in (A) and the temperature in (B) were 7.0 and 37 °C, respectively under static condition. Experiments in (C) were carried out under agitation (100 rpm and 120 rpm) or without agitation (0 rpm) at 37 °C and initial pH 7.0. The decolorization extent was measured after 96 h of cultivation with 200 mg L^−1^ dye and 5% inoculum.

Since the initial dye concentration has strong inhibitory effects on dye decolorization and degradation (Khehra et al., 2005; Kalme et al., 2007), decolorization activity of *B. cereus* AZ27*, A. faecalis* AZ26 and *Bacillus* sp. AZ28 was investigated using different initial concentrations of NSB-G ranging from 50 to 1000 mg L^−1^ (Fig. 3B). It was observed that the decolorization decreased with an increase in NSB-G concentration. However, decolorization extent by the three bacterial isolates was nearly the same up to 200 mg L^−1^ of NSB-G. Maximum decolorization achieved by *B. cereus* AZ27*, A. faecalis* AZ26 and *Bacillus* sp. AZ28 was approximately 93, 92 and 91%, respectively with 200 mg L^−1^ NSB-G. *Bacillus* species were reported to show optimum decolorization (95–97%) when 200 mg L^−1^ of azo direct blue 151 and azo direct red 31 were used (Lalnunhlimi and Krishnaswamy, 2016), whereas, *Alcaligenes faecalis* PMS-1 showed optimum decolorization (100%) with 400 mg L^−1^ reactive orange 13 (Shah et al., 2012). Decolorization significantly decreased with 500 mg L^−1^ of NSB-G and at 1000 mg L^−1^ of NSB-G, decolorization was severely inhibited. Although bacterial growth was not notably decreased with 500 mg L^−1^ of NSB-G, 30–40% of the bacterial growth was inhibited by 1000 mg L^−1^ of NSB-G in nutrient broth (data not shown).

### 3.5 Effects of temperature, pH and agitation on the decolorization of NSB-G

The optimum temperature for maximum dye decolorization by *B. cereus* AZ27*, A. faecalis* AZ26 and *Bacillus* sp. AZ28 was 37 °C, although an appreciable decolorization occurred at 22–30 °C (Fig. 4A). This optimum temperature for decolorization of NSB-G by the three bacterial isolates was in agreement with *Pseudomonas* sp*.* decolorization of malachite green, fast green, brilliant green, congo red and methylene blue (Mali et al., 2000), *Bacillus subtilis* decolorization of fast red (Mona and Yusef, 2008), and *A. faecalis* PMS-1 decolorization of reactive orange 13 (Shah et al., 2012). However, the decolorization extent was only 10% at 45 °C, which might be due to the loss of cell viability or deactivation of the enzymes responsible for decolorization (Çetin and Dönmez, 2006). This result indicated that 30–37 °C might be the best temperature for decolorization activity of the three bacterial isolates.

The maximum level of decolorization of NSB-G by *B. cereus* AZ27*, A. faecalis* AZ26 and *Bacillus* sp. AZ28 was observed at alkaline pH with the peak at pH 8.0 (Fig. 4B). However, significant levels of decolorization by the three bacterial isolates were observed at pH 5.0–7.0. Nevertheless, the decolorization extent of *B. cereus* AZ27 at pH 5.0–7.0 was significantly higher compared to that of *A. faecalis* AZ26 and *Bacillus* sp AZ28. This result indicated that these isolates could decolorize NSB-G within a wide range of pH, suggesting that these three strains are potential organisms for practical bio-treatment of dyeing mill effluents. The optimum pH for decolorization of NSB-G by the bacterial isolates in the present study is comparable with that of other bacterial strains such as *A. faecalis* PMS-1, *Bacillus* spp. and *Enterobacter* sp. (Shah et al., 2012; Lalnunhlimi and Krishnaswamy, 2016). However, the optimum pH for dye decolorization varies from acidic to alkaline condition (Saratale et al., 2011). The pH tolerance of decolorizing bacteria is quite important because reactive azo dyes bind to cotton fibers by addition or substitution mechanisms under alkaline condition (Aksu and Donmez, 2003; Wang et al., 2013).

The effect of oxygen on cell growth and dye reduction is one of the important factors that need to be considered (Pearce et al., 2003). The bacterial isolates showed good growth under agitation (data not shown) but the decolorization of NSB-G was better in static condition (Fig. 4C). In static condition, decolorization was almost 10% more compared to that under agitation. This result indicated that static condition was better for reactive dye decolorization, although the growth of bacteria was less than that under agitated conditions. Similar reports have shown that static condition was necessary for dye decolorization but the growth of bacteria significantly increased under agitation (Khadijah et al., 2009; Jadhav et al., 2011; Wanyonyi et al., 2017; Thanavel et al., 2018). Decolorization under static condition may be attributable to azoreductase that degrades azo bond, having nonspecific oxidation capacity and does not require oxygen as an electron acceptor (Kalyani et al., 2008).

### 3.6 Bacterial decolorization manner of NSB-G

**Fig. 5:**
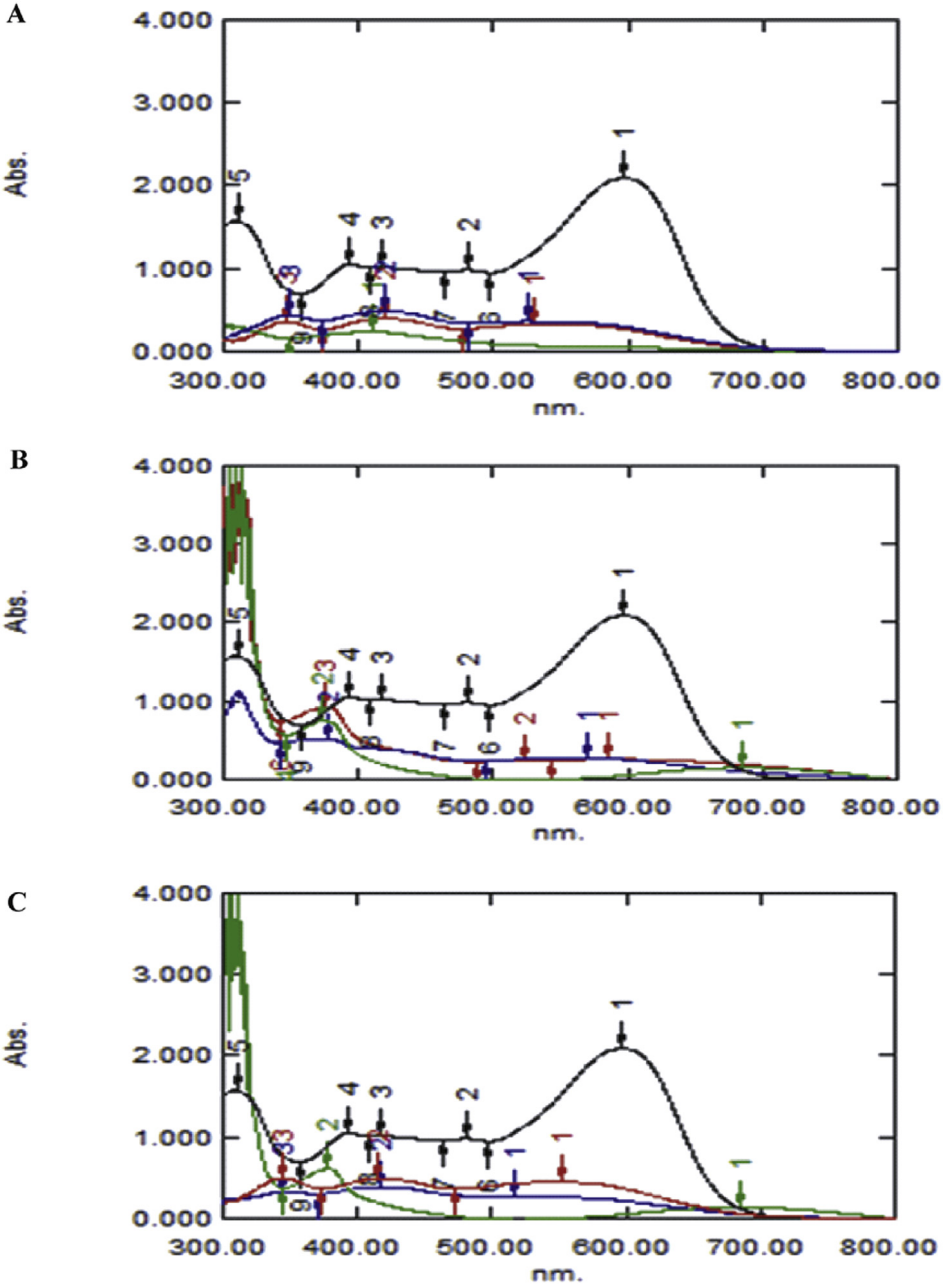
Variation in the UV-vis spectra of NSB -G before and after decolorization by *B. cereus* AZ27 (A)*, A. faecalis* AZ26 (B) and *Bacillus* sp. AZ28 (C). Black, blue, red and green lines indicate decolorization of NSB-G after 0, 24, 48 and 96 h of incubation under optimum conditions.

There are different mechanisms of dye decolorization. It may be by bacterial adsorption (Aravindhan et al., 2007) and/or biodegradation (Kumar et al., 2007; Veena et al., 2019). To understand the manner of decolorization of NSB-G, the supernatant was analyzed by UV-visible spectrophotometer and FTIR. A UV–vis spectral scan (200–800 nm) of the supernatant after 96 h decolorization showed that the maximum absorbance wavelength in visible spectra shifted from 600 nm to 410 nm, 378 nm and 373 nm by *B. cereus* AZ27*, A. faecalis* AZ26 and *Bacillus* sp. AZ28, respectively (Fig. 5). The shift in light absorbance spectra is attributed to new metabolites due to biodegradation of the parent compound (Yu and Wen, 2005; Pandey et al., 2007; Sen et al., 2019). In adsorption, the intensity of all peaks changes approximately in proportion without any new peak or without the shift of absorbance spectra (Veena et al., 2019). The absorbance peak at UV spectra did not disappear in the end of decolorization indicating that NSB-G was not completely mineralized while some new metabolites were formed in the culture supernatant (Barapatre et al., 2017).

**Fig. 6:**
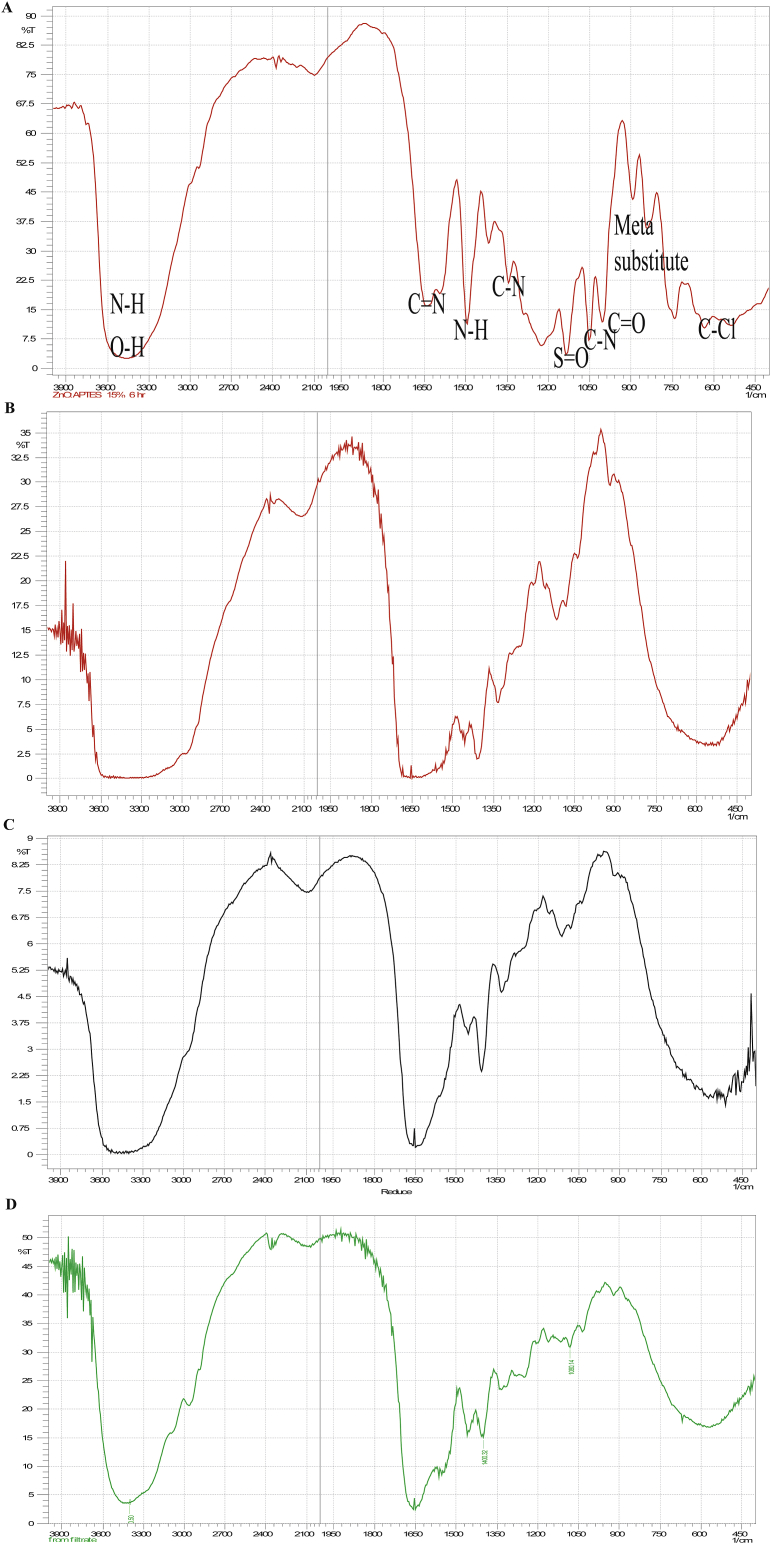
FTIR spectra of parent NSB-G (A) and degraded products of NSB-G by *B. cereus* AZ27 (B)*, A. faecalis* AZ26 (C) and *Bacillus* sp. AZ28 (D).

Biodegradation of NSB-G by bacterial isolates was confirmed by FTIR spectroscopic analysis (Fig. 6)*.* Biodegradation is indicated either by disappearance of absorbance peaks or appearance of new peaks (Chen et al., 2008; Sen et al., 2019). The FTIR spectrum of the parent NSB-G represented the stretching vibrations of S=O at 1134 cm^−1^, C–N at 1051 cm^−1^, C–O at 1004 cm^−1^, C–N at 1342 cm^−1^, C=N at 1639 cm^−1^ and C-Cl at 632 cm^−1^. The FTIR spectrum of metabolites extracted after 48 h showed disappearance of the stretching vibrations stated above and appearance of some new peaks in different positions in comparison with those of the parent NSB-G (Fig. 6). *A. faecalis* AZ26 showed six new major absorption peaks at 873, 931, 1294, 1384, 1429 and 1581 cm^−1^, *B. cereus* AZ27 showed seven new peaks at 881, 943, 1174, 1355, 1382, 1477 and 1652 cm^−1^, and *Bacillus* sp. AZ28 showed nine new peaks at 885, 889, 1020, 1112, 1247, 1344, 1382, 1479 and 1529 cm^−1^. Different FTIR spectrum of parent NSB-G and the extracted metabolites clearly indicated that the biodegradation of the parent NSB-G was occurred by the three bacterial isolates. Disappearance of absorption peaks and appearance of new peaks in the FTIR spectrum were reported due to biodegradation of reactive orange 13 and methyl orange (Shah et al., 2012; Sen et al., 2019).

## 4 Conclusions

Three bacterial isolates having the best capability to decolorize reactive textile dyes were screened and identified as *Alcaligenes faecalis* AZ26, *B. cereus* AZ27 and *Bacillus* sp. AZ28 based on their biochemical characteristics and 16S rDNA sequences. The decolorization of Novacron Super Black G by the bacterial isolates is due to biodegradation and is dependent on various physico-chemical parameters. Degradative and decolorizing activity against various reactive dyes suggests that the bacterial isolates in this study have potential practical application in the biotransformation of various dye effluents. Currently, research is going on to characterize the enzymes, especially azoreductase and peroxidase, of these bacterial isolates.

## Declarations

### Author contribution statement

Md. Zobaidul Hossen: Performed the experiments; Analyzed and interpreted the data; Wrote the paper.

Md. Eleus Hussain, Al Hakim: Performed the experiments; Analyzed and interpreted the data.

Kamrul Islam, Md. Nizam Uddin: Analyzed and interpreted the data.

Abul Kalam Azad: Conceived and designed the experiments; Analyzed and interpreted the data; Contributed reagents, materials, analysis tools or data; Wrote the paper.

### Funding statement

This research did not receive any specific grant from funding agencies in the public, commercial, or not-for-profit sectors.

### Competing interest statement

The authors declare no conflict of interest.

### Additional information

Data associated with this studye (nucleotide sequences) has been deposited in DDBJ and will be found in the DDBJ/EMBL/GenBank nucleotide sequence databases with the accession number LC202626, LC202627 and LC202628 for *Alcaligenes faecalis* AZ26, *Bacillus cereus* AZ27 and *Bacillus* sp. AZ28, respectively
